# Research on AUV Multi-Node Networking Communication Based on Underwater Electric Field CSMA/CA Channel

**DOI:** 10.3390/biomimetics9110653

**Published:** 2024-10-25

**Authors:** Xinglong Feng, Yuzhong Zhang, Ang Gao, Qiao Hu

**Affiliations:** 1 School of Mechanical Engineering, Xi’an Jiaotong University, Xi’an 710049, China; fengxinglong@stu.xjtu.edu.cn (X.F.); zzyxjtu@stu.xjtu.edu.cn (Y.Z.); gao2220699841@stu.xjtu.edu.cn (A.G.); 2 Shaanxi Key Laboratory of Intelligent Robots, Xi’an Jiaotong University, Xi’an 710049, China

**Keywords:** underwater communication, electric field, multi-node networking, CSMA/CA, AUV

## Abstract

To address the issues of high attenuation, weak reception signal, and channel blockage in the current electric field communication of underwater robots, research on autonomous underwater vehicle (AUV) multi-node networking communication based on underwater electric field Carrier Sense Multiple Access with Collision Avoidance (CSMA/CA) channel was conducted. This article, first through simulation, finds that the Optimized Link State Routing (OLSR) protocol has a smaller routing packet delay time and higher reliability compared to the Ad Hoc On-Demand Distance Vector (AODV) protocol on underwater electric field CSMA/CA channels. Then, a 2FSK underwater electric field communication system was established, and dynamic communication experiments were carried out between two AUV nodes. The experimental results showed that within a range of 0 to 3.5 m, this system can achieve underwater dynamic electric field communication with a bit error rate of 0 to 0.628%. Finally, to avoid channel blockage during underwater AUV multi-node communication, this article proposes a dynamic backoff method for AUV multi-node communication based on CSMA/CA. This system can achieve dynamic multi-node communication of underwater electric fields with an error rate ranging from 0 to 0.96%. The research results have engineering application prospects for underwater cluster operations.

## 1. Introduction

The future underwater operation mode is transforming towards intelligence, autonomy, and collaboration. Due to the limitations in size and payload of a single operational platform, its intelligent capabilities are insufficient for complex tasks in battlefield environments. Research on key technologies of underwater AUV multi-node systems provides new ideas for improving future underwater operation modes: Compared to traditional underwater acoustic or optical communication, underwater electric field communication has inherent advantages such as strong anti-interference capability, fast communication rate, and suitability for close-range multi-node communication. An AUV multi-node system composed of multiple platforms can obtain more comprehensive information compared to a single node. Different types of detection devices carried by multiple nodes can enhance the diversity of the multi-node system’s functions, providing guarantees for completing complex tasks. The collaborative network coverage of the cluster system is large and can improve search efficiency by simultaneously obtaining information from multiple nodes. The number of nodes in the underwater multi-node cluster system is flexible, with strong scalability and resistance to failures of a single node, demonstrating high survival and adaptability capabilities.

In recent years, wireless communication networks have been favored by people for their convenience, flexibility, and speed, making people’s lives faster and more colorful. They have been widely used in various scenarios. Among them, ad hoc networks have received increasing attention, and routing protocols, as one of the most important components of ad hoc networks, have become a hot research direction. Vemuri et al. [1] used NS-3.31 simulation to analyze the performance of OLSR, AODV, Destination Sequenced Distance Vector (DSDV), and Dynamic Source Routing (DSR) protocols under different conditions. It was observed that AODV had the highest average reception rate and average packet reception, DSDV consumed the least energy, while DSR consumed the most energy. Shaban et al. [2] compared the performance of AODV, OLSR, and DSDV routing protocols in multi-hop networks and observed the performance of various routing protocols in vehicle scenarios using the NS-3.31 network simulator. Kartheeban et al. [3] proposed two group key establishment and distribution protocols for secure multicast group communication among resource-constrained sensor nodes in the Internet of Things. In addition, using NS-3.31 simulations based on different nodes, the proposed protocols were examined for their relevance based on three different performance parameters: cost, overhead, and packet loss.

Sahu et al. [4] proposed the idea of using cluster tokens to identify clusters, where the sender cluster checks the receiver cluster for identification before initiating any communication. They used the NS-3.31 simulator to simulate and verify their approach. Sharma et al. [5] proposed a wormhole tunnel node communication transmission model and simulated it using the DSR routing protocol in NS-3.31 under wormhole attack scenarios. Chang [6] simulated Ad Hoc networks using the NS-3.31 network simulation tool and implemented the code development and debugging of an improved AODV routing protocol, improving network performance. Han et al. [7] proposed a transmission media access control (MAC) protocol based on Register Transfer Level (RLT) encoding without handshake and optimized the Acknowledge Character (ACK) frame format. The results showed that the optimized protocol can effectively reduce conflicts, improve transmission reliability, and save energy consumption. Mao [8] proposed a dynamic backoff media access control (DB-MAC) protocol with dynamic backoff for handshake, which improves the utilization of underwater acoustic channels and demonstrates better network performance in experiments. Wang [9] proposed the use of the Detection-Based Power Control Routing protocol for network data transmission, which reduces the duration of data collection. Bianchi et al. [10] proposed an adaptive competitive window mechanism that dynamically selects the optimal backoff window. Alvarez et al. [11] proposed a hybrid visible light communication and radio frequency femtocell network access protocol based on the CSMA/CA algorithm. Xie Guangming’s team proposed a CSMA/CA communication protocol to solve the collision problem among multiple robots [12]. Cicioğlu et al. [13] constructed an underwater network environment consisting of a physical layer and a data link layer and analyzed the transmission characteristics of underwater acoustic channels. For underwater robots, static channel allocation is not suitable due to disconnections or joining the network during the deployment process or due to insufficient energy, and there is a problem of wasted channel resources. However, for dynamic media access control, the most typical CSMA/CA protocol has been widely used. Currently, the CSMA/CA protocol has been applied in underwater communication systems and has been verified for its feasibility. Due to the prioritized channel resources and distributed topology structure of the system, the underwater electric field cluster communication system is more suitable for adopting the CSMA/CA protocol.

The complex underwater environment severely attenuates commonly used communication methods in terrestrial communication, making it a technical challenge to ensure smooth information exchange between autonomous underwater vehicles (AUVs). Wang et al. [14] found that various temporal and spatial scales of ocean dynamics seriously affect the reliable detection and decoding of signals by underwater acoustic communication devices. Donati et al. [15] found that underwater optical communication is susceptible to hydrological conditions and scattering of water. However, the discovery of weakly electric fish provides a new approach to integrated underwater group communication [16]. Blouin et al. [17] found that weakly electric fish can emit weak electric fields through their electric organ discharge and sense distorted electric fields on their body surface. The concept of signal transmission through electric field conduction was first proposed by Tucker [18], and the emission and detection processes of electric field communication signals were realized. Momma et al. [19] discovered that the effective distance of electric field communication is a function of current, and the feasibility of electric field communication was verified through experiments. Xie Guangming’s team analyzed the factors affecting communication distance in underwater current field communication [20]. At the same time, the team integrated the underwater electric field communication system into a bionic robotic fish and successfully controlled the fish’s swimming mode through an external emission device, demonstrating the promising application prospects of this communication system in close-range communication of underwater robots [21]. Feng et al. [22] proposed an integrated method for underwater electric field detection and communication based on P4 code modulation OFDM signals to meet the requirements of underwater robot cluster operations. Zhao et al. [23] proposed a dynamic velocity potential field method to enhance the swarm performance for the cooperative hunting task of multi-autonomous underwater vehicles with moving obstacles. Therefore, this article adopts underwater electric field communication for signal transmission, which has good communication omnidirectionality, high speed, and low delay, and meets the requirements of dynamic communication among AUV multi-nodes.

**Figure 1:**
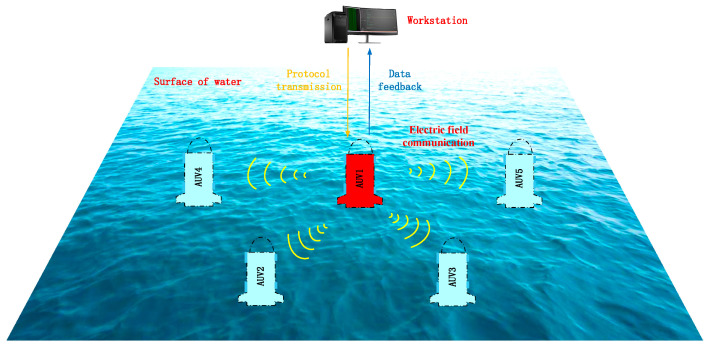
Schematic diagram of AUV multi-node network communication based on the underwater electric field.

This article proposes a novel underwater electric field multi-node networking protocol for the application scenario of AUV collaborative networking. It designs an underwater multi-mobile node electric field communication protocol and channel allocation technique and validates the effectiveness of the protocol through simulation analysis. This research aims to develop communication strategies for underwater electric field multi-node networking, and focuses on the following aspects: conducting simulation parameter design and calculation for electric field communication based on the characteristics of underwater electric field transmission; constructing an NS-3.31 underwater electric field multi-node networking communication network model and setting up simulation models for various layers of the multi-node communication network; conducting simulation program analysis for multi-node networking, comparing the impact of OLSR and AODV protocols on the communication performance of multi-node networking, and evaluating the performance of the simulation communication results; and finally, conducting dynamic communication experiments for the underwater electric field communication system based on AUV, and further conducting dynamic backoff multi-node communication experiments based on AUV using CSMA/CA, to verify the effectiveness of the underwater electric field communication protocol, thus achieving collision-free underwater electric field multi-node communication. The schematic diagram of AUV multi-node network communication based on underwater electric field is shown in Figure 1.

The remaining parts of this article are organized as follows: In Section 2, the setup of the underwater electric field communication simulation model is presented. In Section 3, an analysis of the simulation program based on underwater electric field communication is conducted. In Section 4, the design and experimental verification of the underwater electric field communication channel backoff protocol are completed. Section 5 presents the main conclusions of this article.

## 2. Simulation Model Setting for Underwater Electric Field Communication

In underwater node communication, although MAC protocols applicable to underwater communication networks, both domestically and internationally, consider long propagation delays, node energy efficiency, and transmission efficiency, there are few MAC protocols designed for underwater node mobility. Especially when AUVs participate in networking, the nodes in underwater dynamic self-organizing networks move relatively quickly, resulting in most traditional MAC protocols no longer being applicable. For MAC protocols that require centralized nodes, strict scheduling order, and relatively fixed topology, they cannot support the implementation and deployment of dynamic self-organizing networks. Most communication protocols are mainly applied to underwater acoustic communication, with few targeting underwater electric field communication protocols. The MAC protocol was mainly designed to improve channel utilization efficiency and maximize fairness for channel users, but there is a problem of wasting channel resources in time in underwater environments. There are currently two main underwater channel allocation techniques for electronic communication systems: dynamic media access control and static channel allocation. For underwater robots, static channel allocation is not suitable and there is a problem of channel resource waste due to disconnection or joining the network during deployment or insufficient energy during the movement process; for dynamic media access control, the most typical CSMA/CA protocol has been widely used. This protocol does not allocate channel media to any node, but adopts a random channel allocation method, which can fully utilize the channel. And in order to solve the problem of channel conflicts, the article considers the mobility of underwater electric field communication in multi-node networking, designs a MAC protocol based on CSMA/CA avoidance, and compares the impact of OLSR and AODV routing protocols on node communication performance, thereby ensuring the real-time and reliability of AUV multi-node networking communication.

**Figure 2:**
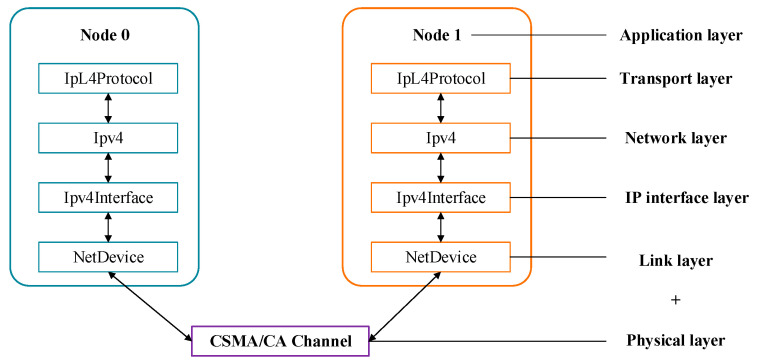
NS-3.31 virtual network structure.

In response to the relatively simple and easy-to-learn, good scalability, and resource-saving characteristics of the NS-3.31, a simulation model for underwater electric field communication based on CSMA/CA channels was constructed, and simulation and research on various levels of networks, protocols, etc., were achieved [24,25]. This article constructs a CSMA/CA underwater electric field communication simulation model with five nodes and verifies the effectiveness of the simulation model through parameters such as packet delivery rate and delay. A computer network is a network where multiple computers are connected through network cards and media, and the machines are equipped with corresponding software protocols. In NS-3.31, the application layer and protocol stack are connected, and the application layer and IP layer are connected through the transport layer. In NS-3.31, the network equipment includes the MAC layer and the physical layer, and the channel is equivalent to the media layer. Among them, hardware includes network nodes, network connection cards, and network connection cables, while software includes TCP/IP network protocol stacks, etc. The CSMA/CA channel network structure constructed in this article includes an application layer, transport layer, network layer, IP layer, link layer, and application layer, achieving the design of the entire multi-node communication simulation process. Thus, the construction of the network simulation environment for nodes, network devices, channels, and applications in NS-3.31 [26] was completed, and the model validation of multi-node network communication in underwater electric field communication was achieved. The NS-3.31 virtual network structure based on CSMA/CA is shown in Figure 2.

The node model in this article sets the initial position coordinates for five nodes using the MobilityHelper object and configuring properties related to “position allocator functionality”. The mobility helper in this node model uses a two-dimensional grid to initially place the nodes. The underwater electric field communication nodes are set within a 60 m × 60 m area, and communication between multiple nodes is established. Next, the nodes are added to a NodeContainer, which is a helper class in NS-3.31 that allows for operations on multiple nodes and stores pointers to these objects in the system. Then, the CAMA/CA channel is installed to connect the electric field communication devices to the nodes, creating a network topology for underwater electric field communication with multiple nodes. After that, the OLSR and AODV routing protocols are installed based on the TCP/IP protocol stack, which installs a corresponding network protocol stack for each node in the node container. Finally, the appropriate application layer protocol is chosen based on the selected transport layer protocol to enable data communication between the server and clients, completing the setup of the entire simulation process.

When AUV multi-nodes communicate in electric fields underwater, they have the advantage of good omnidirectional data parameters, but there is a characteristic of signal transmission attenuation, mainly related to transmission distance. Therefore, in underwater multi-node network communication based on CSMA/CA channels, it is necessary to determine the propagation loss model at a specific distance. Due to the long transmission distance of underwater electric fields in low-frequency communication, the communication frequency, i.e., carrier frequency 14 kHz, is adopted. Next, based on the characteristics of underwater media, a 15 m path loss model is calculated, and the calculation results are imported into the NS-3.31 network model to complete the underwater electric field multi-node networking communication model based on CSMA/CA channels, verifying the stability and backoff characteristics of signal transmission between nodes. The CSMA/CA channel attenuation model will be calculated based on the relationship between underwater communication transmission distance and signal attenuation, providing a theoretical basis for NS-3.31 simulation based on underwater electric field communication.

In underwater electric field communication, the transmission distance is related to the signal attenuation model, which is also known as the propagation model. This model implements the Friis propagation loss model, which was first described in [27]. The original equation was described as follows:

(1) $$\frac{P_{r}}{P_{t}}=\frac{A_{r}A_{t}}{d^{2}\lambda^{2}}$$

where $A_{r}$ is the effective receiving area of the receiving end (m^2^) and $A_{t}$ is the effective transmitting area of the transmitting end (m^2^).

For an isotropic antenna with no heat loss, the following equations are used:

(2) $$A_{t}=\frac{\lambda^{2}}{4\pi }$$

The final equation becomes the following:

(3) $$\frac{P_{r}}{P_{t}}=\frac{\lambda^{2}}{{\left(4\pi d\right)}^{2}}$$

The initial expansion of this original equation is as follows:

(4) $$P_{r}=\frac{P_{t}G_{t}G_{r}\lambda^{2}}{{\left(4\pi d\right)}^{2}L}$$

where $P_{t}$ is the transmit power (W), $P_{r}$ is the receive power (W), $G_{t}$ is the transmit gain, $G_{r}$ is the receive gain, λ is the wavelength (m), d is the distance (m), and L is the system loss.

The conductivity and permeability are set to $\sigma =4\;Sm^{-1}$ and $\mu =1.257\times 10^{-6}\;Hm^{-1}$, respectively. The communication frequency is set as the carrier frequency f=14 kHz.

The wavelength of the propagating electric field in seawater can be expressed as follows:

(5) $$\lambda =\frac{2\pi }{\sqrt{\pi f\mu \sigma }}=13.361\;m$$

In the experiment, wave speed ν=λf, in which f is the frequency measured in Hz, can be configured by the user using the “frequency” attribute.

(6) $$\nu =\lambda f=1.871\times 10^{5}\;m/s$$

The following formula implements the LogDistance propagation model, which is used to calculate received power using the so-called LogDistance propagation model.

(7) $$L=L_{0}+10n\log\left(\frac{d}{d_{0}}\right)$$

where n is the path loss distance exponent, $d_{0}$ is the reference distance (meters), $L_{0}$ is the path loss at the reference distance (dB), d is the distance (meters), and L is the path loss (dB).

The LogDistance model is used in the NS-3.31 simulation. Firstly, the correct reference attenuation value $L_{0}$ is calculated using the Friis model based on the frequency and propagation speed. Then, the attenuation value at any distance can be computed. In this article, the reference attenuation value $L_{0}$ is calculated for the underwater electric field transmission frequencies f=14 kHz, λ=13.361 m, $d_{0}=1\;m$, n=2, and $G_{t}=G_{r}=1$, respectively.

(8) $$L_{0}=-10\log\left(\frac{P_{r}}{P_{t}}\right)=-10\log(\frac{G_{t}G_{r}\lambda^{2}}{{\left(4\pi d_{0}\right)}^{2}})=-0.5326\;dB$$

According to the LogDistancePropagationLossModel, the total loss L for a distance of d=15 m is calculated as follows:

(9) $$L=L_{0}+10n\log\left(\frac{d}{d_{0}}\right)=22.9892\;dB$$

When the transmission power EnergyDetectionThreshold is set to 16.0206 dB, the threshold for the state is as follows:

(10) 16.0206−22.9892=−6.9686 dB

The threshold for the busy state of CCA (Clear Channel Assessment) in CCAModeThreshold is −6.9686 dBm.

In the NS-3.31 simulation parameters based on CSMA/CA underwater electric field multi-node networking, the energy detection threshold is set to −96 dBm, the CCA busy state threshold is set to −99 dBm, and the energy level is set to 1. Based on the calculation of underwater electric field communication parameters, the simulation parameters for underwater electric field communication are established as shown in Table 1.

## 3. Simulation of Underwater Electric Field Communication

### 3.1. Underwater Electric Field Communication Simulation Flow Setup

**Figure 3:**
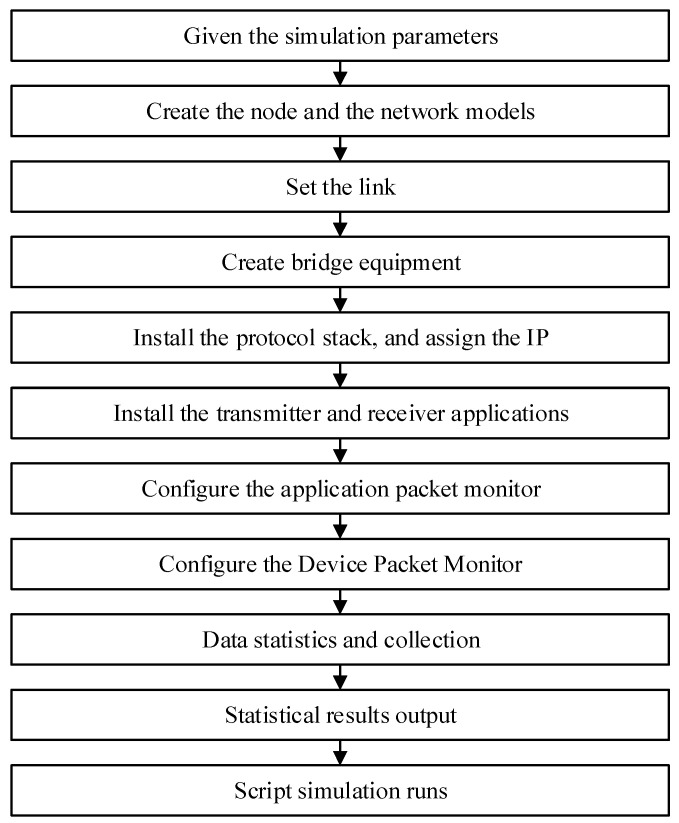
Simulation process of underwater multi-node electric field communication network based on CSMA/CA.

Based on the calculation of underwater electric field communication parameters, a simulation of underwater electric field communication multi-node networking was conducted based on CSMA/CA protocol. The simulation process for the underwater multi-node electric field communication network structure based on CSMA/CA is shown in Figure 3. Firstly, the simulation parameters for the multi-node network structure of underwater electric field communication were given, and the nodes and network models participating in the communication were created by setting up links to connect each node to the network model. Secondly, bridge devices were created, protocol stacks were installed, and IP addresses were allocated to all mobile nodes. Then, transmitter and receiver applications were installed, and the configuration of the application packet monitor and device packet monitor was completed. Finally, the port sending and receiving packets for each node were created, data statistics and collection were completed, and the statistical results were output for the entire script simulation run [28]. The parameters for underwater electric field communication were established as shown in Table 1.

The text describes the installation of a server-side listener on port 9, named echoServer (9). The UdpEchoServerHelper object has an Install method that connects the application to a node. The Install method takes the NodeContainer as a parameter, with an implicit C++ conversion using the result of nodes.Get(1) as the argument. The unnamed NodeContainer is then passed to the Install method. echoServer.Install will install a UdpEchoServerApplication on the node with index 1 in the NodeContainer of the manager node. The installation returns a container that includes pointers to all the applications created by the helper. Similarly, the client-side application is set up using a UdpEchoClientHelper to manage the UdpEchoClientApplication. It sends a 14 kbit UDP packet to port 9 of the node after the simulation starts and stops after 10 s.

By running the NS-3.31-based CSMA/CA visualization interface code via a visualization program, the visualization interface for communication between five nodes of the underwater electric field, Node 0, Node 1, Node 2, Node 3, and Node 4, based on the CSMA/CA protocol in a simulation area of 60 m in length and 60 m in width, can be obtained.

**Figure 4:**
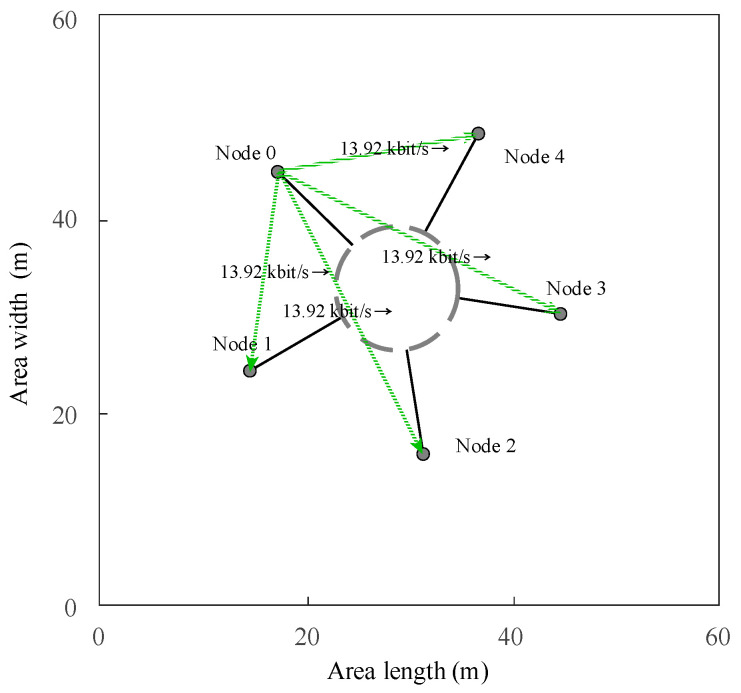
The networking results of underwater electric field communication with multiple nodes.

The NS-3.31 simulator was used to test the simulation output of underwater electric field communication based on CSMA/CA, which included five nodes and underwent 20 simulation runs. The simulation results showed that the same results were obtained for communication data transmission. The simulation results indicated that a data transmission rate of 13.92 kbit/s could be achieved for communication data transfer from one node to four nodes in the CAMA/CA underwater electric field communication channel. The simulation results validate that the NS-3.31 simulation model can realize network communication based on the CSMA/CA protocol for underwater electric field communication, laying the foundation for the subsequent experimental verification of AUV multi-node channel backoff, and the communication result is shown in Figure 4.

### 3.2. Analysis of Underwater Electric Field Communication Simulation Results

Based on the calculation results of the underwater electric field communication simulation parameters, a communication experiment with two nodes at a fixed distance of 15 m was conducted to verify the effective communication range of the two nodes. Finally, a comparative analysis of the communication delay and packet delivery rate of the five nodes under different protocols was performed.

#### 3.2.1. Underwater Electric Field Communication Distance Simulation Testing

The receiving process of target nodes in underwater electric fields is as follows: when a node receives the first bit of a data packet, it checks the received energy. If the energy is below the energy detection threshold for channel transmission, the packet will be discarded. If the energy reaches the corresponding threshold, NS-3.31 ultimately determines the bit error rate and packet loss rate based on the SNIR (Signal to Interference plus Noise Ratio), which determines whether the data packet is successfully received. The LogDistancePropagationLossModel is an extension of the Friis free space model and can be accessed through the following paths with Config::Set and Config::Connect [29,30]. It is used to predict propagation loss in a wide range of environments, while the Friis free space model is limited to unobstructed paths between the transmitter and receiver.

**Figure 5:**
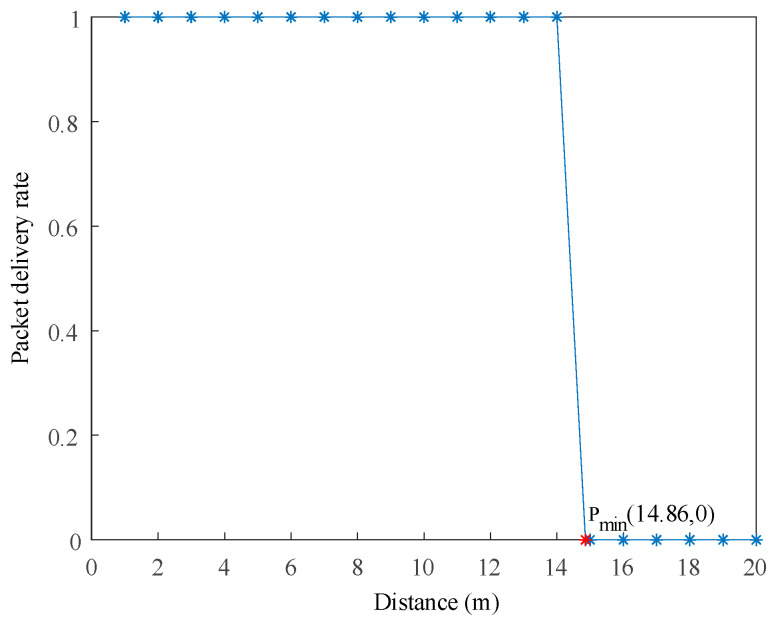
Analysis of underwater electric field communication distance test results.

The NS-3.31 simulation environment was used to evaluate the underwater electric field communication path propagation attenuation process in seawater, using the LogDistancePropagationLossModel. By using this model to calculate the received power, the transmission power was returned when requesting the path loss at a distance less than the reference distance. The correct reference attenuation value was obtained based on the frequency and propagation speed using the Friis model. Then, the attenuation value at any distance was calculated, allowing for communication experiments with two nodes at a fixed distance of 15 m to be conducted. The simulation results are shown in Figure 5.

In the NS-3.31 simulation, the communication process between two nodes was observed by gradually increasing the distance between them by 1 m. The packet delivery rate at the receiving node was monitored, and when the packet delivery rate changed from 1 to 0, it indicated that effective data transmission was not possible at that distance. The communication distance was then gradually decreased to find the maximum communication distance. The simulation results showed that the minimum communication distance, Pmin, was 14.86 m, indicating that the maximum communication distance was within this range. Within the range of 14.86 m, the packet delivery rate for underwater electric field communication was 100%, while beyond this range, the packet delivery rate was 0. Therefore, for future AUV point-to-point dynamic experiments, communication distances within this range should be selected to validate the performance of underwater electric field communication.

#### 3.2.2. Underwater Electric Field Communication Packet Delivery Rate Simulation Test

The underwater electric field was evaluated by using the packet delivery ratio to assess the stability of data transmission in underwater CSMA/CA channel communication. The packet delivery ratio is a rate that measures the proportion of data packets sent and received in a network. In an ideal situation, as many data packets are sent, an equal number of packets should be received. However, due to factors such as signal attenuation and network quality in underwater electric field communication, the ideal scenario does not always occur. The packet delivery ratio is mainly influenced by network traffic and congestion levels in each routing segment from the computer to the website server. Since TCP/IP networks can automatically implement retransmission, packet loss can result in even more packet loss. Therefore, it is common to observe an increasing packet loss rate after network congestion occurs [31,32].

**Figure 6:**
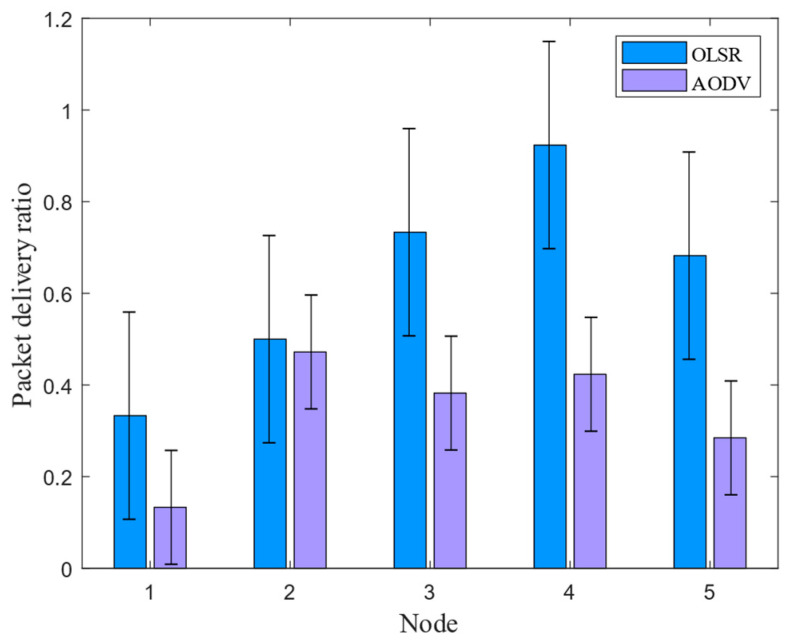
Comparison analysis of packet delivery rate in underwater electric field communication under different protocols.

The packet delivery rate is calculated by the upper layer of the routing layer during data transmission. It represents the ratio between the total number of received packets and the total number of sent packets in the network. This ratio indicates the probability of successful packet delivery in the entire multi-node communication network, as well as the packet loss rate due to link failures during the transmission process. In the simulation of multi-node communication in underwater electric fields, this parameter can effectively reflect the efficiency of the routing protocol in transmitting data between nodes, and the Formula (11) defines this parameter. In this simulation scenario, two protocols (OLSR and ADOV) were applied to five nodes within 10 s. The results of the packet delivery rates of the two routing protocols under the CSMA/CA channel are shown in Figure 6.

(11) $$Packet\_ratio=\frac{d_{Tx}}{d_{Rx}}$$

where Packet_ratio represents the packet delivery rate, $d_{Tx}$ represents the total number of received packets, and $d_{Rx}$ represents the total number of sent packets.

The simulation results show that in the communication process, OLSR had a higher packet reception rate and higher data transmission reliability compared to the AODV routing protocol when five nodes were used. Therefore, in future underwater field communication, it is recommended to use the OLSR routing protocol based on the CSMA/CA channel for multi-node communication. This can improve the stability of signal transmission between nodes and ensure effective underwater multi-node operations.

#### 3.2.3. Underwater Electric Field Communication End-to-End Delay Simulation Test

In underwater electric field communication, end-to-end delay is used to evaluate the smoothness of network transmission in the underwater CSMA/CA channel. End-to-end delay consists of three parts: transmission delay, encapsulation delay, and queuing delay. Transmission delay is determined by the link length divided by the propagation speed, so it is a fixed value that only depends on the link length and transmission speed. Encapsulation delay is the time it takes for the first byte of data to enter the link until the last byte enters the link, which is the time it takes to put the entire packet into the link. Queuing delay occurs when multiple packets arrive at the same router at the same time and are stored in the router’s buffer in order of arrival for transmission. Therefore, this delay depends on how many waiting packets are in front of the data packet, and it is an uncertain delay [33].

End-to-end delay is measured by the routing layer when receiving data, and it represents the average time it takes for a data packet to successfully travel from the source node’s routing layer to the destination node’s routing layer. In underwater electric field communication, this parameter is used to reflect the smoothness of data transmission in the network, where a smaller delay indicates a smoother network. Its definition is shown in Equation (12).

(12) $$d_{end-end}=\frac{d_{TotalTime}}{d_{TotalNumber}}$$

where $d_{end-end}$ is the end-to-end average delay, $d_{TotalTime}$ is the sum of the transmission time of all data packets arriving at the destination node, and $d_{TotalNumber}$ is the number of data packets arriving at the destination node.

**Figure 7:**
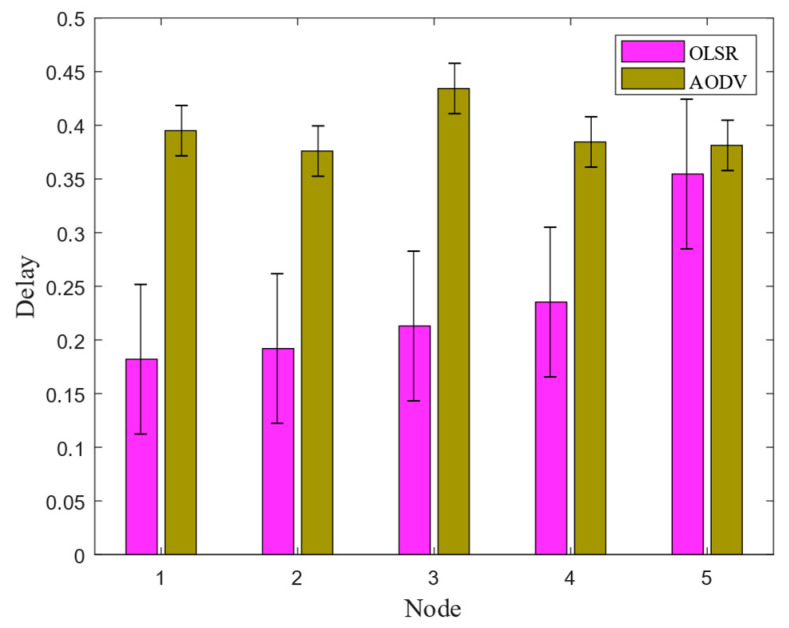
Comparison analysis of underwater electric field communication delay.

In communication networks, the number of packets in the node cache represents the number of packets stored in the routing layer buffer. The fewer the number of packets, the shorter the time the data stays in the wireless router, allowing the data to be forwarded faster. The number of discarded packets due to the lack of routing refers to the number of packets discarded by the node when the corresponding routing response does not arrive before the end of the routing discovery period after the node sends out the routing request. In the simulation scenario, two protocols (OLSR and ADOV) were applied to five nodes within 10 s. The comparison results of the end-to-end delay of the two routing protocols under the CSMA/CA channel are shown in Figure 7.

The simulation results show that the OLSR routing protocol had a smaller routing packet delay and shorter communication time compared to AODV during communication among five nodes. Therefore, in the subsequent underwater electric field communication, OLSR routing protocol based on the CSMA/CA channel was chosen for multi-node underwater communication. This can reduce the time delay in signal transmission between nodes, ensuring real-time operations and improving operational efficiency in underwater multi-node.

Thus, in the subsequent design and experimental verification process of the underwater electric field communication channel contention protocol, the OLSR routing protocol was chosen to be embedded in the CSMA/CA channel design process, ensuring larger received packet size and shorter routing packet delay time in multi-node communication, thereby improving the network topology structure of underwater multi-node clusters, providing guarantee for high reliability and low delay data transmission, as well as improving the signal transmission stability and real-time performance between nodes.

## 4. Design and Experimental Verification of Backoff Protocol for Underwater Electric Field Communication Channel

To achieve the dynamic underwater electric field multi-node communication system, the primary issue to address was the communication problem within the underwater multi-node. Since there was only one underwater communication channel with a large number of communication nodes, the signals emitted by the robots through the electric field would collide and cause coupling in the underwater channel, resulting in error codes. Therefore, this section aims to solve the backoff problem of underwater electric field multi-node communication in the underwater channel.

### 4.1. CSMA/CA Channel Backoff Algorithm Design and Simulation Analysis

The design and simulation process of the CSMA/CA channel backoff algorithm for underwater electric field multi-node communication in underwater channels, targeting the backoff problem, is shown in Algorithm 1.
**Algorithm 1** An algorithm with CSMA/CA backoff protocol** while** *Channel detection is Idle* **do**   *Distributed inter frame spacing***   if** *Channel detection is Busy* **then**    *Random backoff process*
    *Random number(R) selection*
    *Time slot t delay*
**    if** *Channel detection is Idle* **then**     *R = R* − 1 **     if** *R* = 0 **then**
      *Data transmission***     else**       *Time slot t delay*
**     end if****   else**     *Time slot t delay*
**   end if****  else***     Data transmission***  end if**** end while**

The random backoff process in this system is based on the binary exponential backoff algorithm. It involves delaying transmission by randomly selecting a backoff time. During the backoff process, the basic backoff time is defined as a time slot, denoted as t. When an AUV enters the random backoff process, it selects a random backoff number, r, from the discrete integers [0, 1, …, (2k − 1)]. At the end of each time slot, the underwater channel is checked. If the channel is idle, r is decremented by 1; otherwise, r remains unchanged, and the AUV waits for another time slot before continuing to check the underwater channel. After continuously cycling through this process, when r becomes 0, the AUV can send the message. In this system, the DIFS (Distributed Inter-Frame Space) is set to 100 ms, and the time slot t is set to 100 ms.

**Figure 8:**
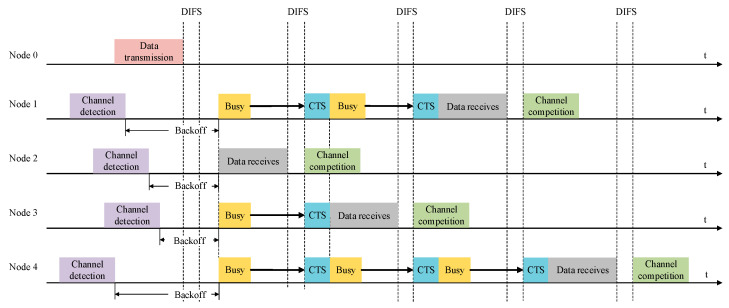
CSMA/CA backoff mechanism between five nodes.

Figure 8 shows that when Node 0 was sending data, Nodes 1, 2, 3, and 4 were receiving underwater electric field communication data. Due to the detection of busy channels by all four nodes, they all needed to perform backoff algorithms and randomly backoff for a period of time before sending the data. From Figure 8, it can be seen that the backoff timer of Node 3 decreased to zero first, and then Node 3 completed the reception of communication data. When Node 3 was receiving data, Nodes 1, 2, and 4 detected that the channel was busy and made data input response requests. The values of their respective backoff timers were set to wait for the channel to become idle again. After Node 3 received the data and the DIFS had passed, the backoff timers of Nodes 1, 2, and 4 started counting down from their respective remaining time. As Node 1, Node 2, and Node 4 were competing for the channel, Node 2’s backoff timer was reduced to zero first, so Node 2 received the data. Subsequently, Nodes 1 and 4 completed the reception of communication data in sequence.

**Figure 9:**
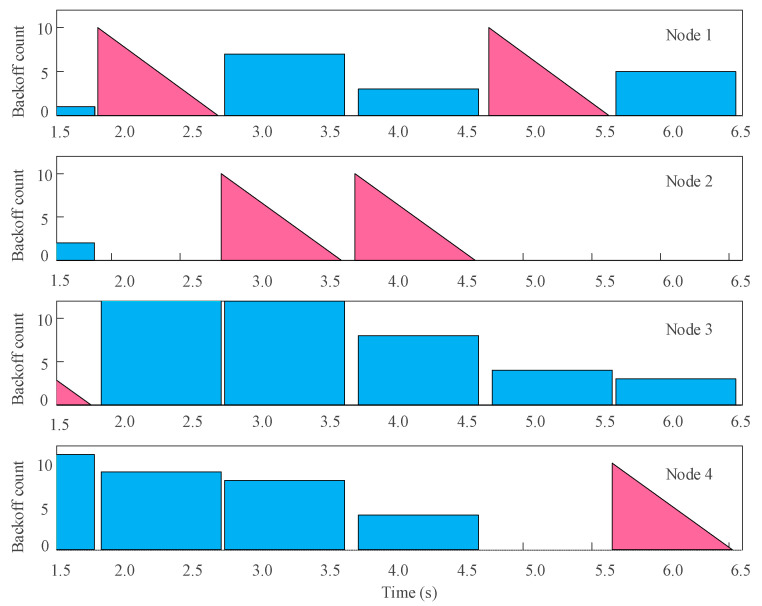
CSMA/CA dynamic backoff process.

To verify the feasibility of the protocol in the AUV multi-node communication system, a simulation analysis of the protocol was first conducted using MATLAB. Figure 9 shows the dynamic backoff process of the protocol. The rectangles represent random backoff numbers, and the triangles represent ongoing transmissions.

The simulation results for the CSMA/CA dynamic backoff protocol algorithm process are as follows:

- At t = 1.85 s, Node 3 generated a communication request and detected an idle channel. After waiting for DIFS, the node started transmitting the entire frame (the first triangle in Figure 3);
- At t = 1.91 s, after Node 3 finished transmitting the signal, the channel became idle again and generated a new data transmission request. Nodes 1, 2, and 4 were in a random wait process, and Node 1, with the lowest random backoff number, started occupying the channel for data transmission;
- At t = 2.58 s, Node 2’s random B number became 0, and the channel was not occupied. Node 2 started sending data;
- At t = 3.72 s, Node 2 requested to occupy the channel again, but after DIFS and two-channel detections, the channel was still not occupied by other nodes. Therefore, Node 2 was allowed to prioritize channel occupancy;
- At t = 4.73 s and t = 5.62 s, Node 1 and Node 4’s random backoff numbers became 0 one after another, and they were allowed to use the channel.

### 4.2. Design and Implementation of Underwater Electric Field Multi-Node Communication System Based on Raspberry Pi

Based on the previous section, it can be inferred that the collision problem in AUV channels can also be solved in the same way using CSMA/CA. Therefore, this article adopts Raspberry Pi for the design and implementation of the CSMA/CA protocol. Currently, Raspberry Pi 4B can be equipped with 4 GB of memory, has four USB ports and a Gigabit Ethernet port, and can be connected to a network cable, mouse, and keyboard. It also features an HDMI video interface, an analog video signal TV interface, and a camera interface with stereo headphone output. All these functions are integrated into a small-sized motherboard, and only a screen and keyboard are required to achieve full PC functionality, such as hardware operation, software programming, video playback, etc. Due to its low price, Raspberry Pi is widely used and greatly improves the portability of the underwater electric field communication system as the main control center [34].

#### 4.2.1. Software Environment Setup

In this study, the Raspberry Pi served as the main control system for AUV multi-node communication, providing the operation of the CSMA/CA backoff protocol and the transmission and reception of communication information. First, the operating system needed to be installed on the Raspberry Pi, and the CSMA/CA backoff algorithm and data transmission and reception modules needed to be developed. The Raspberry Pi officially provides the Raspberry operating system, which is developed based on Linux and specifically designed for the Raspberry Pi, offering enhanced security and greater versatility.

#### 4.2.2. Launch Circuit Excitation Signal

**Figure 10:**
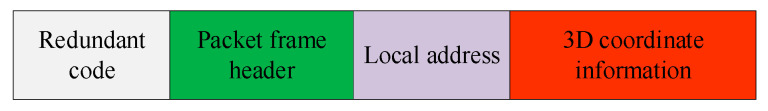
Underwater electrical field communication data packet information.

In underwater electric field multi-node communication systems, the most basic elements are baseband signals and carrier signals. This article proposes using a Raspberry Pi to generate baseband signals and carrier signals. Based on the Raspberry Pi 4B development board, which has six UARTs including four new PL011 UARTs compared to Raspberry Pi 0, 1, 2, and 3’s dual UARTs. Firstly, due to the default closure of the other four UARTs in the Raspberry Pi system, the command “sudo nano/boot/config.txt” is used to configure UART 1 (ttyAMA1), where UART 1 corresponds to GPIO0 and GPIO1 for the read and write IO ports, respectively. By loading the Python-specific Serial library and configuring the UART through the serial.Serial() function, parameters such as baud rate, stop bits, and parity bits are set. The data packet is encoded and sent using serial. write(), and UTF-8 encoding and decoding are used in this article. Since the ultimate goal of this system is to achieve an AUV multi-node, the data packet mainly transmits the self-position coordinates. Other information that needs to be included is the frame header, local address, and redundancy code, as shown in Figure 10. The redundancy code is a single character that aims to adjust the PGA gain to an appropriate amplification factor by sending redundant information in advance to avoid signal distortion when the robot’s position changes in real-time. The frame header is a fixed character “99” used to detect useful information in the data packet.

For the 2FSK communication system, the carrier signal is a 14 KHz PWM wave. In Raspberry Pi, pigpio is a library for controlling the General Purpose Input Output (GPIO). The pigpio library is suitable for all versions of Pi. Software-timed PWM is available on all GPIO 0–31, but since the carrier signal in this system reaches up to 14 kHz and software-timed PWM can only achieve a maximum of 1400 Hz, the hardware PWM of Raspberry Pi needed to be used. Raspberry Pi 4B has four output ports, with BCM12 and 18 as one group, and 13 and 19 as the other group. By calling the pi.hardware_PWM() function to configure the PWM frequency and phase, the hardware PWM of Raspberry Pi can be enabled.

#### 4.2.3. CSMA/CA Channel Backoff Protocol

To solve the potential collision problem in the AUV multi-node, the previous section of this article introduced the design of the CSMA/CA channel avoidance algorithm. This section aims to integrate the CSMA/CA channel avoidance protocol into the Raspberry Pi platform for physical verification. The CSMA/CA protocol flowchart is shown in Algorithm 2. The underwater channel occupancy is identified by checking the Uart_rec_flag channel flag twice. If the channel is occupied, a random backoff time is generated, with the backoff time unit being the data packet transmission time. Once the backoff period is over, the underwater electric field communication baseband signal and carrier signal are generated through the UART and hardware PWM.
**Algorithm 2** An CSMA/CA backoff protocol process** While** (1) **do****   if** *Data_bu f = =* 0 **then**
     while(1)**   else****     if** *Uart_rec_flag = =* 0 **then****      ***Delay(DIFS)***      if** *Uart_rec_flag*! = 0 **then****       ** *Uart_rec_flag* = 0**       ** *T = Random*()        *Delay*(*t*)        **if** *Uart_rec_flag*! = 0 **then****        ***Uart_rec_flag* = 0       **else****        ***T* = *T* − 1**        if** *T* = 0 **then**          *Set_PWM_handware*(18, 12000, 50000)          *Uart_send*(*Data_buf*)          *Set_PWM_handware*(18, 12000, 0)          *Data_buf* = 0          while(1)        **else**          *Delay*(*t*)**        end if****       end if****      else**       *Set_PWM_handware*(18, 12000, 50000)**      end if****     else***      Uart_rec_flag* = 0**     end if****   end if**** end while**

#### 4.2.4. Channel Detection

Before applying the CSMA/CA-based protocol to the electronic communication system, there was another key issue that needed to be addressed—channel state detection. The flowchart of the channel detection algorithm is shown in Algorithm 3. The channel detection algorithm was mainly designed based on the Raspberry Pi serial port. Firstly, the serial library function ser. read (ser. inwaiting()) is used to read all the real-time data from the Raspberry Pi serial port, which is the received data of the underwater current field communication. The Python-specific string library function data_rev.find() is used for string recognition, to detect the frame header of the data packet. When the frame header is detected, it indicates that the underwater channel is occupied, and the channel detection flag is set to 1. Then, combined with the data packet composition in the previous section, the position of the frame header is detected first by the function Data_rev.index(), and then it is determined whether the next byte after the frame header in the data packet is the target address of the local ID. If they are the same, the three-dimensional position coordinates in the data packet are extracted. Otherwise, the next round of loop detection is performed.
**Algorithm 3** An CSMA/CA channel detection process **While** (1) **do**   *Data_rev = ser_read(ser.in Waiting*()))**   if** *Data_rev.find*(“99”) = = −1 **then**    *threadLock.acquire*()     *Uart_rec_flag* = 0    *threadLock.release*()    *Data_rev* = = 0    while(1) **   else**    *threadLock.acquire*()    *Uart_rec_flag* = 1    *threadLock.release*()    *B* = *Data_rev.index*(“99”) **    if** *Data_rev*[*B +* 2] *= = Local I D* **then***     Position = Data_rev*[*B +* 4 *: B +* 13] **    else**     *Data_rev* = = 0**    end if****  end if** **end while**

### 4.3. Underwater Electric Field Dynamic Multi-Node Communication System Water Tank Experiment Verification

#### 4.3.1. Experimental Validation of Underwater Electric Field Mobile Communication Based on AUV

**Figure 11:**
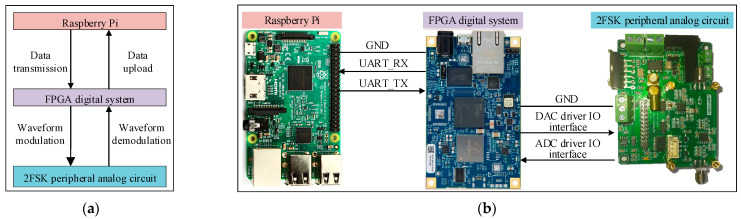
Schematic and physical diagram of the 2FSK underwater electric field communication system. (**a**) Schematic diagram of the experiment. (**b**) Physical system of underwater electric field communication.

In this article, a 2FSK underwater electric field communication system was built and integrated into an AUV for multi-node underwater electric field experimental testing [35,36]. The underwater electric field physical communication system based on 2FSK includes the Raspberry Pi main control board, FPGA digital communication system, and 2FSK peripheral analog circuit, as shown in Figure 11. Raspberry Pi interacts with FPGA digital communication system through the UART interface, and FPGA uses digital-to-analog conversion to output 2FSK modulated waves to the 2FSK analog circuit. The 2FSK analog circuit transmits the underwater-received electric field communication signal to FPGA for processing through the ADC driver.

To achieve a miniaturized and integrated underwater electric field wireless communication system, a communication system combining digital and analog circuits was designed. The transmitting end of the communication system includes a transmitter and a transmitting electrode. The transmitter system can be divided into PC, FPGA, analog-to-digital converter, and power amplifier. The PC upper computer sends raw information to FPGA through the UART communication interface, and combines the received binary signals into decimal information in FPGA for channel encoding. Subsequently, the encoded data are converted into binary signals and frequency shift keying modulation is performed in FPGA. The continuous phase digital signal generated by modulation is converted into a continuous phase analog frequency band signal through a high conversion rate DAC circuit. Finally, the converted analog frequency band signal needs to be power amplified, and the amplified frequency band signal is emitted into the water through a pair of electrodes, forming an electrostatic field in the water.

**Figure 12:**
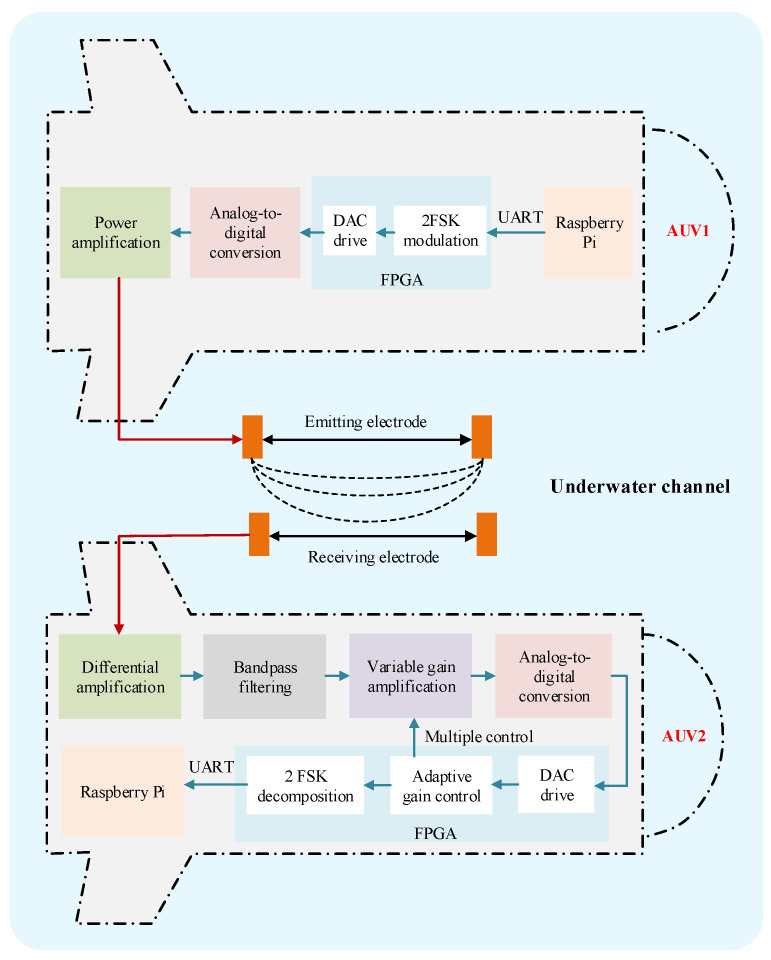
Communication process of AUV based on 2FSK.

In this article, an underwater electric field communication system based on 2FSK was built, and data transmission between underwater AUVs was completed, laying the foundation for AUV multi-node communication. The main process is shown below. Firstly, AUV1 sends communication data through Raspberry Pi, which undergoes 2FSK modulation, analog-to-digital conversion, and power amplification before being transmitted to the underwater channel through the transmitting electrode. Then, the AUV2 receiving the electrode processes the received communication data through differential amplification, bandpass filtering, variable gain amplification, and digital-to-analog conversion into the FPGA for processing, completing the signal’s DAC drive, adaptive gain control, and 2FSK envelope demodulation. Finally, it is uploaded to the Raspberry Pi to complete the entire communication process. The communication process of AUV is based on 2FSK, as shown in Figure 12.

**Figure 13:**
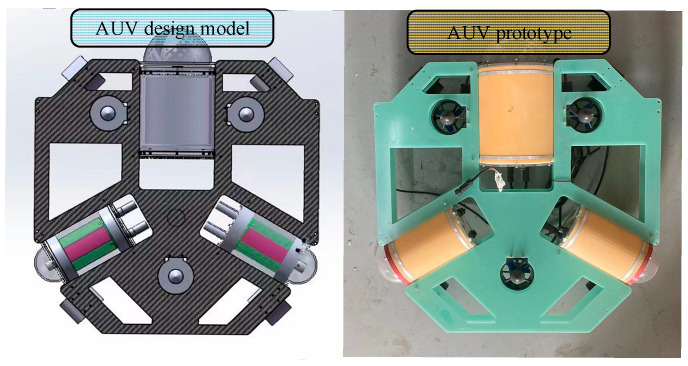
AUV based on 2FSK communication system.

To verify the feasibility of underwater dynamic communication for AUVs, this section details the experiments conducted on AUV underwater electric field dynamic communication. As the AUV swarm moves during the process of tracking and capturing, it is necessary to achieve dynamic mobile communication for the underwater electric field. Therefore, this study experimented on underwater electric field dynamic communication, as shown in Figure 13, where the transmitting and receiving electrode plates and the 2FSK underwater electric field communication system are installed on two AUVs.

**Figure 14:**
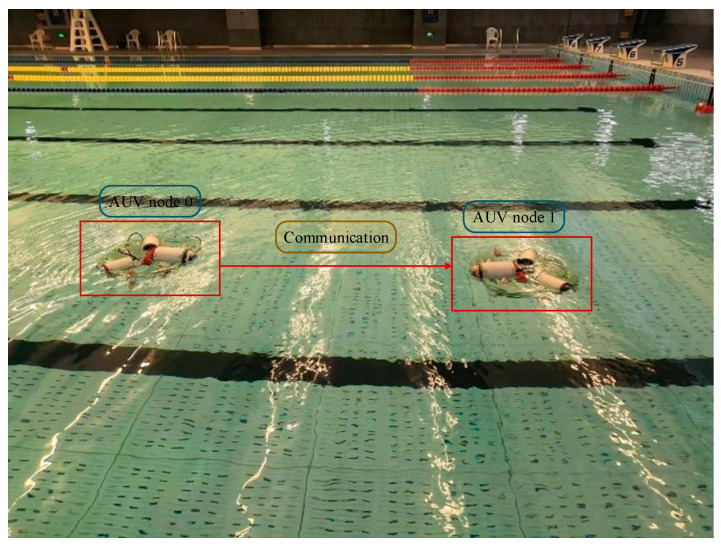
Underwater electric field point-to-point dynamic communication experiment.

The communication distance is changed by moving the two AUVs. In this process, the receiving end of the PGA amplification circuit adjusts the amplification factor through the FPGA program to amplify the received signal voltage to the appropriate level to meet the demodulation requirements. The PC Raspberry Pi remote control software is used to view the demodulation information, as shown in Figure 14.

**Figure 15:**
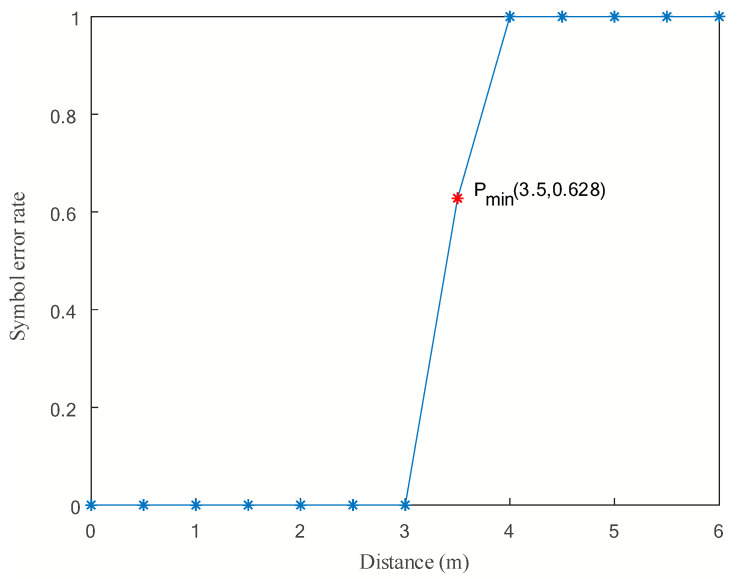
AUV point-to-point underwater dynamic electric field communication.

The point-to-point dynamic communication experiment of the underwater electric field was conducted in a water tank with dimensions of 50 m in length and 25 m in width. The purpose of the experiment was to verify the effectiveness of underwater electric field communication for mobile AUVs by adjusting the relative distance between AUV Node 0 and AUV Node 1. The experimental results of AUV point-to-point underwater dynamic electric field communication are shown in Figure 15.

During the experiment, the distance between two AUV nodes for communication was increased by 0.5 m at each interval, while keeping the two AUVs in a relatively low-speed dynamic state, to observe the bit error rate of the speed dynamic state, and observe the bit error rate of the receiving node. When the bit error rate changed from 0 to 1, it indicated that effective data transmission was not possible at the current point, thus determining the maximum communication distance.

The experimental results show that the minimum communication distance Pmin was 3.5 m, indicating the farthest communication distance. The results demonstrate that the underwater electric field communication system based on 2FSK modulation and demodulation can achieve mobile underwater electric field communication with a bit error rate of 0~0.628% within the communication distance range of 0 to 3.5 m. This is because, during the process of AUV movement, the communication distance changed, and the PGA amplification factor was able to achieve error-free communication within a certain communication distance range. However, when the PGA amplification factor needed to be adjusted, some bit errors may have occurred due to the slow convergence speed of adaptive feedback control of the PGA amplification factor. This experiment also validated the feasibility of the designed mobile underwater electric field communication system.

#### 4.3.2. Experimental Verification of Underwater Electric Field Multi-Node Networking Communication Based on AUV

**Figure 16:**
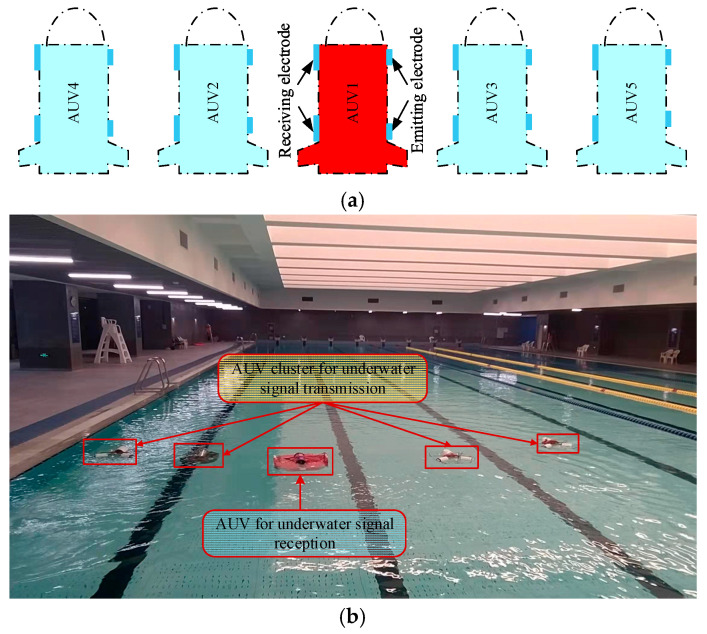
Illustration and experimental diagram of multi-node backoff based on CSMA/CA. (**a**) Schematic diagram of multi-node communication and backoff based on CSMA/CA. (**b**) Experimental diagram of multi-node communication and backoff based on CSMA/CA.

To address the issue of channel conflict during the communication process of underwater electric field AUV multi-node, where multiple nodes simultaneously emit signals, resulting in the distortion of the underwater electric field, distribution, and increased bit error rate, a design and implementation of a multi-node communication system for underwater electric fields was conducted. The CSMA/CA channel backoff algorithm was proposed to solve the channel conflict problem. Dynamic backoff experiments based on CSMA/CA were conducted to verify the feasibility of this algorithm. The experimental scenario is shown in Figure 16a, where the red AUV serves as the receiving node, and the blue AUVs serve as the transmitting nodes. Four transmitting nodes surround the receiving node and perform parallel advancement to conduct multi-node backoff experiments.

This experiment was conducted in a pool environment, as shown in Figure 16b. During the experiment, as each AUV emitted its unique identification code (ID), it was still possible to check which one emitted the electric field communication signal in the experimental results. This article sets the IDs of five AUV Nodes 1, 2, 3, 4, and 5 as 0 × 01, 0 × 02, 0 × 03, 0 × 04, and 0 × 05, respectively. AUV2, AUV3, AUV4, and AUV5 cyclically sent electric field communication information with their IDs to AUV1. During this process, four AUVs competed for the CSMA/CA channel simultaneously, waited, and sent the information according to the proposed mechanism. All transmitted data were monitored by the receiving electrode of AUV1 and sent to the computer through an external receiving and transmitting unit connected to the receiving electrode. By identifying the ID, the specific AUV transmitting the communication data was determined, completing the real-time dynamic backoff process of the entire AUV multi-node.

It can be seen that the AUV can accurately receive the electric field communication information with other AUV identity information. Due to the Raspberry Pi’s adoption of dual-thread synchronous operation of the CSMA/CA backoff algorithm and channel detection algorithm, AUV Node 1 detected that the channel was occupied during communication, entered the backoff process, which is consistent with the simulation and analysis of the underwater electric field communication channel backoff protocol, verifying the accuracy of the integration of the CSMA/CA backoff algorithm and channel detection algorithm in the underwater multi-node communication system designed in this article.

**Figure 17:**
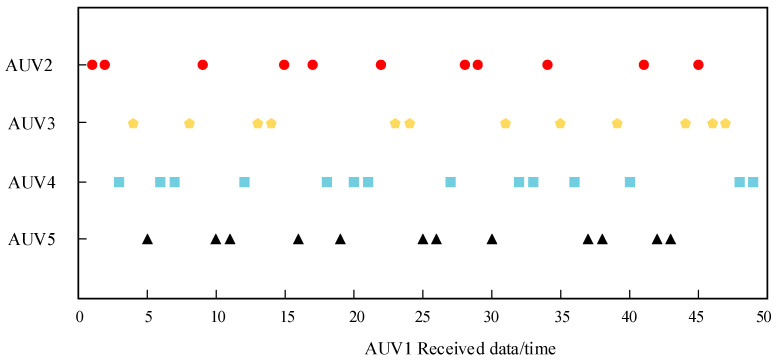
Experimental results of dynamic backoff based on CSMA/CA.

The experimental results based on CSMA/CA dynamic backoff are shown in Figure 17, where the *x*-axis represents the number of data received by the receiving AUV, and the *y*-axis represents the transmitting AUV nodes. Different transmitting nodes emitted signals at different times, and the same receiving node received signals from different transmitting nodes and decoded them to obtain the corresponding coordinates.

From the experimental results, it can be seen that the AUV running the CSMA/CA-based electric field communication protocol can communicate well without communication collisions or conflicts, which still proves the effectiveness of the protocol. This article measured the performance of the underwater electric field multi-node communication system designed in this article based on the bit error rate of 50 other nodes’ underwater electric field communication packets received by AUV1. Through analysis and calculation, it can be concluded that this system can achieve underwater electric field dynamic multi-node communication with a bit error rate ranging from 0% to 0.96%, indicating a low bit error rate. This was mainly caused by the changing communication distances between robots in the multi-node AUV. During the experiment, when the AUV cluster moved forward parallel to each other, the transmitting and receiving electrodes remained parallel, resulting in a bit error rate of 0. However, when the AUV moves at a certain direction angle, the angle between the transmitting and receiving electrode plates constantly changed, leading to a change in bit error rate. Subsequently, the stability of the AUV itself can be increased to reduce the bit error rate. However, based on the experimental results, it can be observed that there was no channel collision and large-scale garbled code generation between nodes during underwater communication, thus verifying the effectiveness of the CSMA/CA backoff protocol designed in this article.

## 5. Conclusions

This article mainly researches the AUV multi-node networking communication based on underwater electric field CSMA/CA channel. In the NS-3.31 simulation process, the propagation physical layer of electric field communication in seawater medium was theoretically calculated, and the program parameters of nodes, channels, network devices, and topology structure were set. The results showed that effective communication could be achieved within a range of 14.86 m between two nodes. The transmission characteristics of five nodes under different protocols were analyzed, and the results showed that the OLSR protocol had a smaller routing reception packet delay time and higher reliability than the AODV protocol in a 60 m× 60 m area. Then, in response to the signal distortion caused by underwater attenuation, a 2FSK underwater electric field communication system was established, and dynamic communication experiments were conducted between two AUV nodes. The experimental results show that the system can achieve underwater dynamic electric field communication within the range of 0 to 3.5 m, with an error rate of 0 to 0.628%. Finally, to avoid channel blockage in underwater AUV multi-node communication, this article proposes a dynamic backoff method for AUV multi-node communication based on CSMA/CA. The experimental results show that the protocol is effective in the process of four transmitters and one receiver for underwater electric field communication, and can achieve collision-free underwater electric field multi-node communication. This system can achieve dynamic multi-node communication of underwater electric fields with an error rate ranging from 0 to 0.96%. The research results have broad engineering application prospects for underwater multi-node cluster operations.

In the future, the authors will explore the influencing factors of target features on achieving precise detection operations and information exchange between underwater swarm vehicles in underwater environments; study the corresponding relationship between active biomimetic detection and communication of underwater electric fields in theoretical modeling; conduct research on the distance-influencing factors of target features on active electric field detection signals and electric field communication; design underwater active electric field biomimetic electrode arrays and underwater electric field communication protocols; and develop an integrated prototype of underwater active electric field detection and communication based on the inherent connections between the detection system and communication system in terms of working principles, system structure, signal processing, etc., and conduct experimental verification.

## Figures and Tables

**Table 1:** Simulation parameters.

Parameter	Value
Simulation environment	NS-3.31 (version3.31)
Operating system	GNU/Linux (Ubuntu18.04.4)
Simulation region	60 m×60 m
Conductivity	$4\;Sm^{-1}$
Permeability	$1.257\times 10^{-6}\;Hm^{-1}$
Communication frequency	14 kHz
Transmission speed	$1.871\times 10^{5}\;m/s$
Number of nodes	5
Propagation loss model	LogDistancePropagationLossModel
Propagation delay model	ConstantSpeedPropagationDelayModel
CCAModeThreshold	−6.9686 dBm
Transmitting power	16.0206 dBm
Channel	CSMA/CA
Transport protocol	OLSR, AODV
Simulation time	10 s
Transmission rate	14 kbit/s

## Data Availability

Data is contained within the article.
